# The association of *RANTES *polymorphism with severe acute respiratory syndrome in Hong Kong and Beijing Chinese

**DOI:** 10.1186/1471-2334-7-50

**Published:** 2007-06-01

**Authors:** Man Wai Ng, Gangqiao Zhou, Wai Po Chong, Loretta Wing Yan Lee, Helen Ka Wai Law, Hongxing Zhang, Wilfred Hing Sang Wong, Susanna Fung Shan Fok, Yun Zhai, Raymond WH Yung, Eudora Y Chow, Ka Leung Au, Eric YT Chan, Wilina Lim, JS Malik Peiris, Fuchu He, Yu Lung Lau

**Affiliations:** 1 Department of Paediatrics and Adolescent Medicine, The Hong Kong Jockey Club Research Centre, Li Ka Shing Faculty of Medicine, The University of Hong Kong, Pokfulam, Hong Kong SAR, China; 2 The State Key Laboratory of Proteomics, Beijing Proteome Research Center, Beijing Institute of Radiation Medicine, Beijing, China; 3 Department of Pathology, Pamela Nethersole Youde Hospital, Hong Kong SAR, China; 4 Department of Pathology, United Christian Hospital, Hong Kong SAR, China; 5 Princess Margaret Hospital, Hong Kong SAR, China; 6 Queen Mary Hospital, The University of Hong Kong, Hong Kong SAR, China; 7 Government Virus Unit, Department of Health, Hong Kong SAR, China; 8 Department of Microbiology, The Hong Kong Jockey Club Research Centre, Li Ka Shing Faculty of Medicine, Pokfulam, The University of Hong Kong, Hong Kong SAR, China

## Abstract

**Background:**

Chemokines play important roles in inflammation and antiviral action. We examined whether polymorphisms of *RANTES, IP-10 *and *Mig *affect the susceptibility to and outcome of severe acute respiratory syndrome (SARS).

**Methods:**

We tested the polymorphisms of *RANTES, IP-10 *and *Mig *for their associations with SARS in 495 Hong Kong Chinese SARS patients and 578 controls. Then we tried to confirm the results in 356 Beijing Chinese SARS patients and 367 controls.

**Results:**

*RANTES *-28 G allele was associated with SARS susceptibility in Hong Kong Chinese (*P *< 0.0001, OR = 2.80, 95%CI:2.11–3.71). Individuals with *RANTES *-28 CG and GG genotypes had a 3.28-fold (95%CI:2.32–4.64) and 3.06-fold (95%CI:1.47–6.39) increased risk of developing SARS respectively (*P *< 0.0001). This -28 G allele conferred risk of death in a gene-dosage dependent manner (*P *= 0.014) with CG and GG individuals having a 2.12-fold (95% CI: 1.11–4.06) and 4.01-fold (95% CI: 1.30–12.4) increased risk. For the replication of *RANTES *data in Beijing Chinese, the -28 G allele was not associated with susceptibility to SARS. However, -28 CG (OR = 4.27, 95%CI:1.64–11.1) and GG (OR = 3.34, 95%CI:0.37–30.7) were associated with admission to intensive care units or death due to SARS (*P *= 0.011).

**Conclusion:**

*RANTES *-28 G allele plays a role in the pathogenesis of SARS.

## Background

Severe acute respiratory syndrome (SARS) is an infectious disease caused by SARS coronavirus [1] with >8000 cases and 774 deaths reported in 2003 [2]. Pathogenesis of SARS is complex and host genetic background is one of the factors in determining susceptibility and outcome [3]. We have demonstrated that genetic haplotypes associated with low serum mannose-binding lectin (MBL) are associated with SARS[4] and our findings were confirmed in another independent study [5]. We furthermore showed that the interferon gamma gene polymorphism, +874 A/T, is associated with SARS [6]. Other susceptibility genes, such as liver/lymph node-specific ICAM-3-grabbing nonintegrin (L-SIGN) which is encoded by *CLEC4M*, 2',5'-oligoadenylate synthetase 1 gene (*OAS-1*) and myxovirus resistance 1 (*MxA*) were also identified [7,8].

Chemokines play important role in cells trafficking during immune responses. Acute respiratory viruses commonly induce inflammatory chemokines in local tissue [9]. In the case of SARS, our previous study confirmed that SARS coronavirus induces upregulation of a number of inflammatoemokines, i.e. Regulated upon Activation, Normal T cell-Expressed and Secreted (RANTES), interferon-gamma inducible protein 10 (IP-10) and Monocyte Chemoattractant Protein-1 (MCP-1) [10]. The upregulation of these chemokines recruit inflammatory cells and leukocytes into the tissue [11]. Therefore, we hypothesized that the polymorphisms of inflammatory chemokine genes might be associated with SARS.

In this study, we investigated the single nucleotide polymorphisms (SNPs) of inflammatory chemokine genes, i.e. *RANTES*, *IP-10 *and monokine induced by gamma interferon gene (*Mig*) in two Chinese cohorts from Hong Kong and Beijing and found that the *RANTES *-28 G allele was associated with disease susceptibility and severity of SARS.

## Methods

### Patients

The study included 495 Hong Kong Chinese patients with SARS. The mean ± SD age was 40.74 ± 15.73 years with 211 males and 284 females (Table 1). At least 95% of the patients were documented with SARS-CoV antibody seroconversion and/or detectable SARS-CoV RNA in respiratory secretions by RT-PCR as described previously in our studies [4,6]. Approval for the study was granted by the Clinical Research Ethics Committee of the Institutional Review Board of the University of Hong Kong/Hospital Authority Hong Kong West Cluster. The patients were further divided into two groups, the death group and the survival group. The death group consisted of 57 patients who died from SARS and their mean ± SD age was 56.2 ± 15.3 years, with 33 males and 24 females. The survival group consisted of 438 patients and their mean ± SD age was 38.7 ± 14.6 years, with 178 males and 260 females. A population of Hong Kong Chinese comprising 578 healthy Red Cross blood donors served as the control subjects. Their mean ± SD age was 30.05 ± 9.49 years, with 343 males and 235 females (Table 1).

**Table 1:** The demographic characteristics of SARSs patients and healthy controls in Hong Kong and Beijing Chinese

	Hong Kong		Beijing	
	Control (n = 578)	SARS (n = 495)	Control (n = 367)	SARS (n = 356)

Sex (male:female)	343:235	211:284	200:156	200:167
Age (mean ± SD)	30.05 ± 9.49	40.74 ± 15.73	34.85 ± 13.5	32.98 ± 12.8

Three hundred and fifty six Beijing Chinese patients with SARS were recruited as described previously (mean ± SD age = 34.85 ± 13.5, 200 male and 156 female) (Table 1) [5]. Among them, 20 patients were classified as severe group, which were identified by their admissions to intensive care units or deaths from SARS (mean ± SD age = 39.45 ± 12.8, 11 male and 9 female). The remaining 336 patients were classified as mild group (mean ± SD age = 34.57 ± 13.5, 189 male and 147 female). A total of 367 ethnically matched healthy individuals (mean age ± SD = 32.98 ± 12.8, 200 male and 167 female) served as controls (Table 1).

### Genotyping

*RANTES *-28C/G (rs2107538) was genotyped by polymerase chain reaction-restriction fragment length polymorphism (PCR-RFLP) as described previously [12].

*RANTES *-403A/G (rs2107538)*, RANTES *In1.1T/C (rs2280789)*, IP-10 *nt1811A/G (rs8878)*, IP-10 *nt2867C/A (rs4859587) and *Mig *nt367A/G (rs10336) were genotyped by the MassARRAY system (Sequenom, San Diego, CA). In brief, the samples were amplified in a 6 μL reaction mixture, containing 5 ng genomic DNA, 0.3 pmol each of specific forward and reverse primers, 200 μL of each dNTP, 3.25 mM MgCl_2 _and 0.2 units of HotStarTaq polymerase (Qiagen, Valencia, CA). PCR conditions included initial hot-start for 15 min at 95°C, 45 cycles of amplification (20 sec at 95°C, 30 sec at 56°C and 1 min at 72°C) and final extension for 3 min at 72°C. The PCR products were treated with alkaline phosphatase to dephosphorylate residual amplification nucleotides. A mixture of 0.2 μL of hME buffer, 0.3 μL of shrimp alkaline phosphatase (1 unit/μL, Sequenom) and 1.5 μL of ddH_2_O was added to the PCR products. The reaction solutions were incubated for 20 min at 37°C, followed by 5 min at 85°C to inactivate the enzyme. Mass-extend reactions to determine genotypes were performed in four groups of different terminations according to the design rationale (ddACG, ddACT, ddAGT and ddCGT, respectively). The reaction volume was 10 μL including 1 unit of Thermosequenase (Sequenom), 50 μM of the respective termination mix, and 0.6 pmol of each assay specific extension primer (Table 2). All assays were run with the same thermal cycle conditions: initial denaturation for 2 min at 94°C followed by 55 cycles of extension (5 sec at 94°C, 5 sec at 52°C and 5 sec at 72°C). Products of the mass-extend reactions were desalted and transferred onto a SpectroCHIP by a nanoliter dispenser according to the manufacturer's instructions (Sequenom).

**Table 2:** Primers used for *RANTES, IP-10, Mig *polymorphisms genotyping by Sequenome

Polymorphisms	Primers (5' to 3')
*RANTES -403*	Forward ACGTTGGATGCTGAGTCTTCAAAGTTCCTG
	Reverse ACGTTGGATGAACATCCTTCCATGGATGAG
	Extension CATGGATGAGGGAAAGGAG
*RANTES In1.1*	Forward ACGTTGGATGCACTCAGTGAACACCTGTAG
	Reverse ACGTTGGATGTGCTTCATGGCAGGGATCTC
	Extension TCTCCTGATCAGTTTTTCTGTCTT
*IP-10 nt1811*	Forward ACGTTGGATGTGGTTGAAAAAAGCAACCCC
	Reverse ACGTTGGATGTAACTGAGCTTTCCTGCTGC
	Extension CCTGCTGCTATGCATTC
*IP-10 nt2867*	Forward ACGTTGGATGTCAACCATGAAAGACTTGGG
	Reverse ACGTTGGATGACCCTGATTACCAGTCAACC
	Extension TCAACCTTGAAGTACAGCTATAAC
*Mig nt367*	Forward ACGTTGGATGTGTAGGAGAGGTTGTCTGTG
	Reverse ACGTTGGATGGCACTCTAAATCATCAGCAG
	Extension TCATCAGCAGTGTGAGC

Genotype determination was performed on a MALDI-TOF mass spectrometer (Sequenom). Mass spectrometric data were automatically imported into the SpectroTYPER (Sequenom) database for data analysis including noise normalization and peak area analysis. The expected molecular weights of all relevant peaks were calculated by the MassARRAY AssayDesign Software (Sequenom) before the analysis and identified from the mass spectrum. In every 96-well plate for assay, there is one well for blank control and five wells for duplicate check on five samples for internal quality control.

### Statistical Analysis

A two-step analysis was used to determine the association of polymorphisms with SARS. The genotype frequencies and allele frequencies of all the genes were compared between SARS patients and controls by a 3 × 2 chi-square test and a 2 × 2 chi-square test respectively, then logistic regression was used for calculating odds ratios (95% confidence interval) and corresponding *P*-values of different genotype frequencies among SARS patients and controls by adjusting for age, sex and all significant single nucleotide polymorphisms (SNPs). Association with the outcomes of SARS infection (death vs survival) was then tested by comparing the genotype frequencies and allele frequencies of all the genes between the death group and the survival group of SARS patients by a 3 × 2 chi-square test and a 2 × 2 chi-square test respectively. The genotype frequencies of all the SNPs were tested for Hardy-Weinberg equilibrium (HWE) separately in SARS patients and controls by chi-square test. Significant *P*-value for multiple testing was adjusted with Bonferroni's correction and all statistical analysis was performed by SAS, version 8.02 and SAS/Genetics (SAS Institute Inc., NC, USA).

## Results

### RANTES -28C/G associated with SARS susceptibility in Hong Kong Chinese

*RANTES *-28C/G,*RANTES *-403A/G, *RANTES *In1.1T/C, *IP-10 *nt1811A/G, *IP-10 *nt2867C/A and *Mig *nt367A/G were genotyped in all 495 SARS patients and 578 controls from Hong Kong. Their genotype and allele frequencies were shown in Table 3 and 4 respectively.

**Table 3:** Genotype frequencies in Hong Kong Chinese patients and controls*

SNP	Control (n = 578)	SARS (n = 495)	OR (95% CI)	*P*
	Number (%)			
**Genotype**				
*RANTES -403*				NS
AA	56 (9.7)	54 (10.9)		
AG	262 (45.3)	223 (45.0)		
GG	260 (45.0)	218 (44.0)		
*RANTES -28*				<0.0001
CC	491 (84.9)	316 (63.8)	Reference	
CG	73 (12.6)	154 (31.1)	3.28 (2.32 – 4.64)	
GG	14 (2.4)	25 (5.0)	3.06 (1.47 – 6.39)	
*RANTES ln1.1*				NS
CC	54 (9.3)	54 (10.9)		
CT	257 (44.5)	217 (43.8)		
TT	267 (46.2)	224 (45.3)		
*IP-10 nt1811*				NS
AA	0 (0)	1 (0.2)		
AG	29 (5.0)	38 (7.7)		
GG	549 (95.0)	456 (92.1)		
*IP-10 nt2867*				NS
AA	0 (0)	1 (0.2)		
AC	31 (5.4)	38 (7.7)		
CC	547 (94.6)	456 (92.1)		
*Mig nt367*				NS
AA	0 (0)	1 (0.2)		
AG	34 (5.9)	38 (7.7)		
GG	544 (94.1)	456 (92.1)		

NS = not significant.

*P-value and OR (95% CI) were calculated with the use of logistic regression models, adjusted with sex and age. After correction by Bonferroni method, the significant *P *value should be less than 0.007

**Table 4:** Allele frequencies in Hong Kong Chinese patients and controls*

SNP	Control (n = 578)	SARS (n = 495)	OR (95% CI)	*P*
	Number (%)			
**Allele**				
*RANTES -403*				NS
A	374 (32.4)	331 (33.4)		
G	782 (67.7)	659 (66.6)		
*RANTES -28*				<0.0001
C	1055 (91.3)	786 (79.4)		
G	101 (8.7)	204 (20.6)	2.80 (2.11 – 3.71)	
*RANTES ln1.1*				NS
C	365 (31.6)	325 (32.9)		
T	791 (68.4)	665 (67.2)		
*IP-10 nt1811*				NS
A	29 (2.5)	40 (4.0)		
G	1127 (97.5)	950 (96.0)		
*IP-10 nt2867*				NS
A	31 (2.7)	40 (4.0)		
C	1125 (97.3)	950 (96.0)		
*Mig nt367*				NS
A	34 (2.9)	40 (4.1)		
G	1122 (97.1)	938 (95.9)		

NS = not significant.

*P-value and OR (95% CI) were calculated with the use of logistic regression models, adjusted with sex and age. After correction by Bonferroni method, the significant *P *value should be less than 0.007

*RANTES *-28 CG and GG genotypes were significantly associated with SARS susceptibility with OR of 3.28 (95% CI: 2.32–4.64) and 3.06 (95% CI: 1.47–6.39) respectively (*P *< 0.0001) (Table 3). *RANTES *-28 G allele was also significantly increased in the patients (*P *< 0.0001, OR = 2.80, 95%CI: 2.11–3.71) (Table 4). After correction by Bonferroni method, the significant *P *value should be less than 0.007, the association of *RANTES *-28C/G to SARS susceptibility remained to have significance.

All genotype frequencies of the six polymorphisms in SARS patients and controls were in HWE except for the *RANTES *-28C/G in controls. To confirm that there is no genotyping error that may contribute to the observation in HWE, direct DNA sequencing was performed in 20–30 samples for each SNP and no ambiguous results were obtained.

### RANTES -28C/G associated with death of Hong Kong Chinese patients

We then compared the genotype and allele frequencies of the *RANTES *-28C/G between the death group and survival group of the SARS patients. *RANTES *-28 G allele associated with death from SARS in a gene-dosage dependent manner (*P *= 0.014), with -28 CG and GG individuals having a 2.12-fold (95% CI: 1.11–4.06) and 4.01-fold (95% CI: 1.30:12.4) increased risk of death from SARS respectively (Table 5).

**Table 5:** Genotype and allele frequencies of *RANTES *-28C/G among death and survival groups in Hong Kong Chinese*

	Death (n= 57)	Survival (n = 438)	OR (95% CI)	*P*
	Number (%)			
*RANTES *-28C/G				
**Genotype**				0.014
CC	26 (45.6)	290 (66.2)	Reference	
CG	25 (43.9)	129 (29.5)	2.12 (1.11 – 4.06)	
GG	6 (10.5)	19 (4.3)	4.01 (1.30 – 12.4)	
**Allele**				0.002
C	77 (67.5)	709 (80.9)		
G	37 (32.5)	167 (19.1)	2.10 (1.30 – 3.39)	

NS = not significant.

*P*-value and OR (95% CI) were calculated with the use of logistic regression models, adjusted with sex and age.

### Association of RANTES -28 C/G with SARS in Beijing Chinese

To further confirm the association of *RANTES *with SARS, we studied the three *RANTES *SNPs in a Beijing Chinese cohort [14]. Three hundred and fifty six SARS patients and 367 healthy controls were genotyped and their genotype and allele frequencies were shown in (Table 6). All genotype distributions of the two groups were in HWE. No significant difference was observed between the frequencies in the SNPs between patients and controls.

**Table 6:** Genotype and allele frequencies of *RANTES *-28C/G in Beijing Chinese patients and controls*

SNP	Control (n = 367)	SARS (n = 356)	OR (95% CI)	*P*
	Number (%)			
**Genotype**				
*RANTES -403*				NS
AA	60 (16.4)	62 (17.4)		
AG	160 (43.6)	153 (43.0)		
GG	147 (40.1)	141 (39.6)		
*RANTES -28*				NS
CC	273 (74.4)	258 (72.5)		
CG	83 (22.6)	87 (24.4)		
GG	11 (3.0)	11 (3.1)		
*RANTES ln1.1*				NS
CC	54 (14.7)	63 (17.7)		
CT	162 (44.1)	152 (42.7)		
TT	151 (41.1)	141 (39.6)		
**Allele**				
*RANTES -403*				NS
A	280 (38.2)	277 (38.9)		
G	454 (61.9)	435 (61.1)		
*RANTES -28*				NS
C	629 (85.7)	603 (84.7)		
G	105 (14.3)	109 (15.3)		
*RANTES ln1.1*				NS
C	270 (36.8)	278 (39.0)		
T	464 (63.2)	434 (60.9)		

NS = not significant.

*P*-value and OR (95% CI) were calculated with the use of logistic regression models, adjusted with sex and age.

Next, we investigated the association of *RANTES *-28C/G with SARS severity. Twenty patients were classified as severe group as defined by their admissions to intensive care units or deaths due to SARS [5]. The genotype and allele frequencies of this SNP in severe and mild patients were shown in Table 7. The genotype distribution among the two groups were significantly different (*P *= 0.011). The frequencies of CG and GG genotypes were over-represented in the severe group (CG: OR = 4.27, 95%CI: 1.64–11.1; GG: OR = 3.34, 95%CI: 0.37–30.7). The frequency of G allele was also significantly increased in the severe group (OR = 2.78, 95%CI: 1.37–5.63, *P *= 0.005).

**Table 7:** Genotype and allele frequencies of *RANTES *-28C/G among severe and mild groups in Beijing Chinese*

	Severe (n = 20)	Mild (n = 336)	OR (95% CI)	*P*
	Number (%)			
*RANTES *-28C/G				
**Genotype**				0.011
CC	8 (40.0)	250 (74.4)	Reference	
CG	11 (55.0)	76 (22.6)	4.27 (1.64 – 11.1)	
GG	1 (5.0)	10 (2.98)	3.34 (0.37 – 30.7)	
**Allele**				0.005
C	27 (67.5)	576 (85.7)		
G	13 (32.5)	96 (14.3)	2.78 (1.37 – 5.63)	

NS = not significant.

*P*-value and OR (95% CI) were calculated with the use of logistic regression models, adjusted with sex and age.

## Discussion

We described here that Hong Kong Chinese with *RANTES *-28CG and GG genotypes had a 3.28-fold (95% CI: 2.32–4.64) and 3.06-fold (95% CI: 1.47–6.39) increased risk of developing SARS respectively (*P *< 0.0001) (Table 3). This -28 G allele also increased the risk of death of Hong Kong Chinese patients with SARS in a gene-dosage dependent manner (*P *= 0.014) that -28 CG and GG individuals had a 2.12-fold (95% CI: 1.11–4.06) and 4.01-fold (95% CI: 1.30:12.4) increased risk (Table 5). More importantly, we further confirmed the association of *RANTES *-28 C/G with the severity of SARS by studying the Beijing Chinese cohort and found that Beijing Chinese patients with CG (OR = 4.27, 95%CI: 1.64–11.1) and GG (OR = 3.34, 95%CI: 0.37–30.7) genotype were more severe, as defined by admission to intensive care units or deaths due to SARS. To further investigate the association of *RANTES *with SARS, we have also performed haplotype analysis using the 3 studied SNPs of *RANTES*, i.e. -403A/G, -28C/G and In1.1T/C, for constructing the haplotypes. However, the major effect of the haplotypes was due to the SNP *RANTES -28 *only (data not shown).

RANTES is responsible for the recruitment of eosinophils, lymphocytes, monocytes and basophils at the site of inflammation and is involved in many viral infections [13,14]. We found that -28 G allele of *RANTES *associated with the susceptibility to and death from SARS. Indeed, *RANTES *-28C/G is located at the NF-κB binding site, which is confirmed by gel-mobility shift assays [15], meaning that this SNP may be involved in the regulation of RANTES expression. Further *in vitro *studies show that *RANTES *-28 G allele enhances NF-κB binding that leads to elevation of promoter activity and increases RANTES expression in CD8+ T cells, CD4+ T cells and monocytes/macrophages [15,16]. Therefore, together with our observation that -28 G allele associated with SARS, we conclude a high level of RANTES may predispose to developing SARS.

This study showed that *RANTES *-28 G allele was a risk factor that associated with severe clinical outcomes in both Hong Kong and Beijing Chinese SARS patients. It has to be noted that many cytokines/chemokines released from activated immune cells not only take part in the process of anti-viral immune response, but are also involved in cell damage and organ dysfunction [17]. Apart from the chemokine receptor signaling pathway, RANTES could activate T cells through herbimycin A-sensitive protein tyrosine kinase (PTK)-mediated pathway at high concentration [18]. This triggers the release of inflammatory cytokines and chemokines such as IL-2, IL-5, IFN-γ and MIP1-β [18]. We have recently demonstrated SARS coronavirus can induce high level of expression of chemokines from human dendritic cells [10]. Too high a level of RANTES may intensify lung inflammation and lead to lymphopenia, increasing the chance of secondary infection and hence death rate among SARS patients [18,19]. Therefore, we speculate that the -28 G allele that associates with the higher level of RANTES may enhance the inflammation and lead to severe clinical outcomes of SARS. Indeed, *RANTES *-28 G allele did show a strong association with death in Hong Kong Chinese patients with SARS (Table 5) and this observation was confirmed in Beijing Chinese that the *RANTES *-28 G allele was associated with admission to intensive care units or deaths due to SARS (Table 7).

In the case of SARS susceptibility, the *RANTES *-28 G allele was associated with Hong Kong Chinese patients only but not in Beijing Chinese patients. It has been suggested that Chinese in southern and northern China may be genetically distinct [20,21], accounting for the different observations with regard to SARS susceptibility.

## Conclusion

We demonstrated that the *RANTES *-28 G allele, which correlates with high RANTES production, was associated with SARS susceptibility in Hong Kong Chinese. It was also associated with adverse outcomes from SARS in both Hong Kong and Beijing Chinese. These suggest that a high RANTES level may play a role in the pathogenesis of SARS.

## Competing interests

The authors do not have any commercial or other association that might pose a conflict of interest.

## Authors' contributions

MWN and GZ: Genotyping, data analyses, drafting the manuscript

WPC: Data analyses, drafting the manuscript

LWYL, HZ, SFSF, YZ: genotyping

HKWL, RWHY, EYC, KLA, EYTC: Sample collection, revising for medical content

WL and JSMP: Sample collection, providing virological data

YLL: Study design, conception and co-ordination, drafting the manuscript

All authors contributed to writing of the final manuscript

All authors read and approved the final manuscript

## Pre-publication history

The pre-publication history for this paper can be accessed here:


